# Meckel’s diverticulum presenting as acute abdomen

**DOI:** 10.11604/pamj.2023.45.54.39639

**Published:** 2023-05-23

**Authors:** Kiran Mastud, Yashwant Lamture

**Affiliations:** 1 Department of General Surgery, Jawaharlal Nehru Medical College, Sawangi, Wardha, India

**Keywords:** Meckel’s diverticulum, obstruction, adhesion

## Image in medicine

A 34-year-old female presented to us in the emergency room with acute abdomen since 2 days. Patient had tachycardia, fever, persistent vomiting, and complaint of not being able to pass stool for 2 days and flatus since 1 day. Per abdomen examination revealed a distended, tender abdomen with absent bowel sounds in all quadrants. X-ray erect abdomen was done which revealed multiple air fluid levels (more than 6) confirming the diagnosis of acute intestinal obstruction. Patient underwent an emergency exploratory laparotomy and was found to have a transition point from a mesodiverticular adhesion causing proximal dilatation of small bowel loop. A diagnosis of small bowel obstruction due to Meckel's diverticulum was made. Adhesion band was released, resection of Meckel's diverticulum and functional end-to-end anastomosis was performed. Meckel's diverticulum is a true diverticulum, containing all layers of the small bowel wall. It is failure of the vitelline duct to obliterate completely, which is usually located on the antimesenteric border of the ileum. It occurs in 2% of the general population and majority of patients remain asymptomatic. When it presents symptomatically it causes painless gastrointestinal bleeding. Nevertheless, in rare instances, it can cause acute intestinal obstruction as it did in our case. The average length of a Meckel´s diverticulum is 3 cm, with 90% ranging between 1 cm and 10 cm and the longest being 100 cm. Our case presents a large Meckel’s diverticulum and the size of diverticulum was 4x2cm.

**Figure 1:**
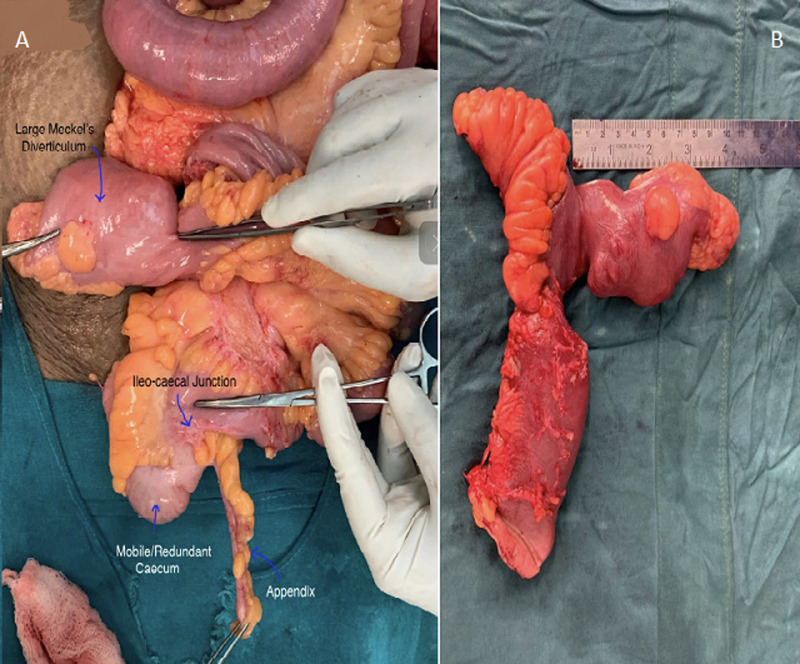
A,B) intraoperative and postoperative picture of Meckel's diverticulum

